# In vitro and in vivo effects of rat kidney vascular endothelial cells on osteogenesis of rat bone marrow mesenchymal stem cells growing on polylactide-glycoli acid (PLGA) scaffolds

**DOI:** 10.1186/1475-925X-6-41

**Published:** 2007-11-04

**Authors:** Hongchen Sun, Zhe Qu, Ying Guo, Guangxiang Zang, Bai Yang

**Affiliations:** 1 Department of Oral Pathology, School of Stomatology, JiLin University, ChangChun, 130041, P. R. China; 2 Department of Oral and Maxillofacial Surgery, Dalian Stomatological Hospital, Dalian, 116021, P. R. China; 3 Key Lab for Supramolecular Structure and Materials of Ministry of Education, College of Chemistry, Jilin University, Changchun, 130012, P. R. China

## Abstract

It is well established that vascularization is critical for osteogenesis. However, adequate vascularization also remains one of the major challenges in tissue engineering of bone. This problem is further accentuated in regeneration of large volume of tissue. Although a complex process, vascularization involves reciprocal regulation and functional interaction between endothelial and osteoblast-like cells during osteogenesis. This prompted us to investigate the possibility of producing bone tissue both in vitro and ectopically in vivo using vascular endothelial cells because we hypothesized that the direct contact or interaction between vascular endothelial cells and bone marrow mesenchymal stem cells are of benefit to osteogenesis in vitro and in vivo. For that purpose we co-cultured rat bone marrow mesenchymal stem cells (MSC) and kidney vascular endothelial cells (VEC) with polylactide-glycolic acid scaffolds. In vitro experiments using alkaline phosphatase and osteocalcin assays demonstrated the proliferation and differentiation of MSC into osteoblast-like cells, especially the direct contact between VEC and MSC. In addition, histochemical analysis with CD31 and von-Willebrand factor staining showed that VEC retained their endothelial characteristics. In vivo implantation of MSC and VEC co-cultures into rat's muscle resulted in pre-vascular network-like structure established by the VEC in the PLGA. These structures developed into vascularized tissue, and increased the amount and size of the new bone compared to the control group (p < 0.05). These results suggest that the vascular endothelial cells could efficiently stimulate the in vitro proliferation and differentiation of osteoblast-like cells and promote osteogenesis in vivo by the direct contact or interaction with the MSC. This technique for optimal regeneration of bone should be further investigated.

## Introduction

Bone deficiency following trauma, resection of tumour, periodontal disease or congenital malformation can be associated with functional and aesthetic problems. To address these issues and to improve patients' well-being bone tissue engineering has been proposed [1-4]. Tissue engineering techniques have mainly been applied on avascular tissue or on other tissue that can grow without an additional vascular supply, such as epithelia, cartilage or large vessel substitutes [5,6]. However, one of the major challenges in regeneration of bone tissue is its vascularization because the center necrosis of the engineered bone tissue will occur if blood supply (nutrition and oxygen) cannot be established quickly [7]. Since diffusion of oxygen in the active tissue is limited about 150 μm from capillary (mean of intercapillary distance (ICD) was 304 ± 30 μm.)[8], vascularization becomes crucial in larger volume of tissue-engineered construct. Growth factors, such as vascular endothelial growth factor (VEGF), collagen type II, myometrial prostaglandin E2, epithelial growth factor and basic fibroblast growth factor (bFGF), have been widely used to accelerate neovascularization in order to regenerate damaged tissues [9,10]. Previously, in vivo secondary vascularization of engineered tissue was attempted with partial success [11]. Alternatively, in vitro construction of vascular stroma could serve as a scaffold for soft or hard-tissue transplant.

Reciprocal regulation and functional interaction between endothelial and osteoblast-like cells during osteogenesis has been reported [1-4,11,12]. Villars et al suggested that membrane proteins as well as systemic hormones and growth factors have an active role in this process [12]. Therefore, to transplant large volume of engineered bone tissue successfully, vascularized bone tissue with the endothelial cells in three-dimensional scaffold in vitro could be used [13]. This may not only solve the nutrition and oxygen diffusion to the middle of the bone tissue [14], but also stimulate osteogenesis by the endothelial cells.

Although some of previous studies showed that vascular endothelial cells and growth factors of vascular endothelial cells could play a role in osteogenesis, it still didn't document well if the direct contact or interaction could be the best way to stimulate osteogenesis, especially in vivo. We hypothesized that the direct contact or interaction between vascular endothelial cells and bone marrow mesenchymal stem cells could be an optimal way to stimulate osteogenesis in vitro and in vivo. Therefore, our objective of present studies was to know what kind of interaction the vascular endothelial cells could efficiently stimulate osteogenesis in vitro and in vivo. To achieve our objective, rat kidney vascular endothelial cells (VEC) and bone marrow mesenchymal stem cells (MSC) were cultured together or alone on PLGA scaffold. The in vitro effect of endothelial cells on osteogenesis by MSC was evaluated. Additionally, MSC-plated PLGA or MSC and VEC-plated PLGA were implanted into the rat's thigh and bone formation was evaluated by soft X-ray analysis and histologically. Our results demonstrated the dramatically effects on osteogenesis in vitro and in vivo while the vascular endothelial cells directly contacted or interacted with the bone marrow mesenchymal stem cells on PLGA scaffold.

## Materials and methods

### Isolation and culture of rat MSC and VEC

Animal experiments were approved by the Animal Care and Use Committee of Jilin University. Male Wistar rats (250 – 350 g, 6–8 weeks old) were anesthetized with intramuscular administration of ketamine (60 mg/kg) and xylazine (8 mg/kg). Bone marrow mesenchymal stem cells were sterilely harvested from the femur and grown in 199 medium supplemented with 10^-6 ^M desacortone, 50 μg/ml ascorbic acid (Invitrogen), 1% L-glutamine, 10% fetal bovine serum (Invitrogen), 100 U/ml penicillin G (Invitrogen), 100 μg/ml streptomycin (Invitrogene, Carlsbad, CA, USA). Osteogenetic potential and calcium precipitation were evaluated by alkaline phosphatase (ALP)[15] and von Kossa [13] staining, respectively. Rats' kidneys were harvested, rinsed with PBS, and their cortex dissected and sliced to 1–2 mm pieces. The tissue was then digested for 2 hours with 0.25% collagenase (type II, Sigma, St. Louis, MO, USA). Disperesed kidney vascular endothelial cells (VEC) were collected and cultured in endothelial growth medium (Technoclone, Austria). CD31 and von-Willebrand factors staining were performed using the ImmunoCruz Staining System (Santa Cruz Biotechnology, Inc., Santa Cruz, CA, USA)[16]. All cultures were grown at 37°C in a humidified 5% CO_2 _atmosphere. After the second passage cells were observed with transmission electron microscopy (HITACHI, Japan)[17].

### Fabrication of Scaffold

Polylactide-glycolic acid (PLGA) was synthesized using a similar approach described by previous reports [18-20]. Briefly, 85:15 (mol:mol) copolymer of D,L-lactide and glycolide (PLG) was milled and sieved to particles ranging from 106 to 250 μm. The PLGA particles were mixed with 5 g NaCl and molded to form a disc (1 cm × 1 cm × 0.5 cm) which was pressured at 800 Pa and under CO_2 _to form an interconnected polymer network. The disc was immersed in ddH_2_O for 24 hour to leach NaCl which resulted in a 95% porus scaffold with 25–400 μm pores. The disc was sterilized with gamma irradiation (Co-60) without effects of physical and chemical characteristics.

### In vitro three-dimensional co-culture models

MSC and VEC were cultured either with direct contact, indirect contact, or separately. (1) In the direct contact setup MSC and VEC (10^4 ^cells/well) were plated on a pre-wetted PLGA disc in a 24-well plate. Two hours later growth medium was added onto the cells. (2) In the indirect contact setup MSC were cultured for 12 hours on pre-wetted PLGA disc as described and then moved onto cultured VEC in 24-well plates. (3) In the third setup MSC and VEC were cultured separately onto PLGA discs as described above [21]. All cultures were grown for 5 days with daily medium changes and then supernatant and cells were harvested for osteocalcin and ALP assays. Protein was quantitated using BCA protein assay kit (Pierce, USA).

### In vivo rat's thigh model

Male Wistar rats (250 – 350 g, 6–8 weeks old) were anesthetized and their bilateral thigh areas were disinfected and incised to expose and separate muscle fibers. PLGA plated with either MSC and VEC or MSC alone and cultured for 2 days were implanted into the right and left thigh muscles, respectively. Skin incisions were sutured and animals were administered a daily intramuscular injection of Benzylpenicillin (10,000 U/day). 8 or 12 weeks following surgery the implants were harvested and subjected to soft X-ray examination and subsequent image analysis (NIH image software, National Institutes of Health, USA) for determination of osteogeneic activity. In addition, samples were stained with hematoxylin & eosin and evaluated histologically.

### Determination of ALP activity and osteocalcin synthesis

The ALP activity was determined using alkaline phosphatase detection kit (Sigma, St. Louis, MO, USA)[13]. Data were expressed as a ratio of unit (U) inorganic phosphate (Pi) enzymatically-cleaved in 30 min/mg protein. Osteocalcin synthesis was determined using osteocalcin radioactivity kit (Biosource, Germany)[19].

### Statistical analysis

All experiments were repeated 6 times. Data were analysed with student *t*-test and multiple range test (SPSS 11.5, Chicago, USA) and presented as mean ± standard deviation (SD). Differences were considered significant when *p *< 0.05.

## Results

### Evaluation of MSC and VEC cultures

In the presence of desacortone and ascorbic acid and after 3 passages the morphology of MSC changed from fusiform monolayer to multilateral shaped, multilayer small colonies. The cells demonstrated even higher proliferation rate after 4 or 5 passages. After 7 days, a ALP-positive black zone could be seen around the cells and von-Kossa-positive calcium deposition was noted. These results indicate that MSC posses osteoblast-like characteristics. VEC were CD31 and von-Willebrand-positive and had a round and oval (stone-like) morphology arranged as a road array (data not shown).

### In vitro effect of VECs on MSCs osteogenic potential in the three-dimensional co-culture

**Figure 1:**
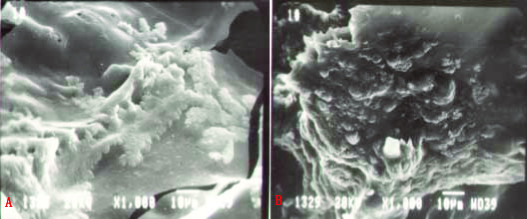
Image of the MSCs and VECs on the PLGA surface from scanning electron microscopy. A. MSCs adhere and extend on the PLGA surface. The arrow indicates the pseudopodium (× 1000). B. VECs adhere on the PLGA surface as an oval and stone-like shape (× 1000).

**Figure 2:**
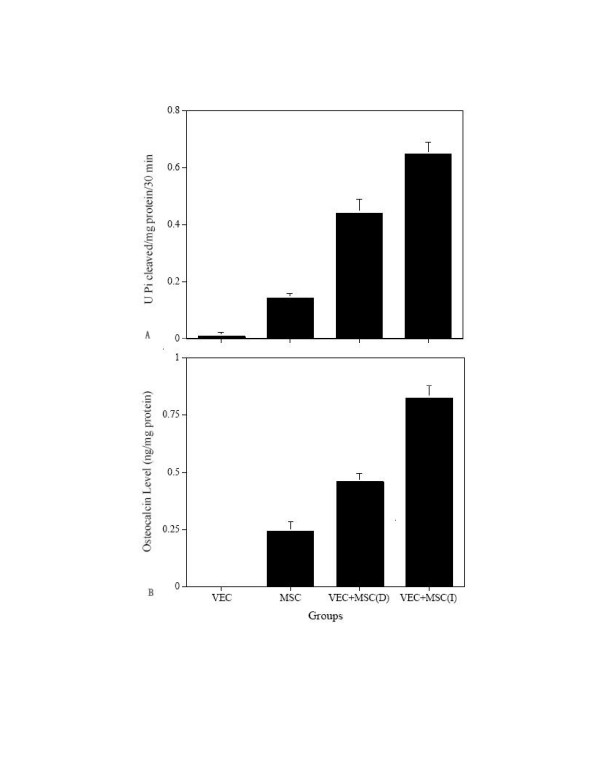
ALP activity and osteocalcin synthesis from three-dimensional co-culture in vitro. A. The ALP activity. B. Osteocalcin synthesis. The data shown are the means ± SD from six experiments. (I) means indirect. (D) means direct.

Five days after culturing MSC and VEC on PLGA the discs were examined by scanning electron microscopy and the results are presented in figure 1. MSC could be seen adhered and extended on the PLGA surface with pseudopodium present between the cells (Fig. 1A). Oval and stone-like shaped VEC forming prevascular network-like structures were also seen on the PLGA surface (Fig. 1B). The osteogenic potential of the 3 culture setups on PLGA was measured using ALP activity and osteocalcin synthesis and the results are displayed in figure 2. Cultured MSC had low ALP activity of 0.15 ± 0.02 whereas indirect-contract co-cultures of MSC and VEC had a higher activity of 0.44 ± 0.05. Significant (p < 0.05) increase in ALP activity (0.65 ± 0.04 U/mg protein) was seen in direct contact co-cultured MSC and VEC setup (Fig. 2A). Synthesis of osteocalcin (a specific marker of bone cells) showed similar results to those of ALP and are shown in Figure 2B. Direct contact co-cultures of MSC and VEC had a significantly (p < 0.05) higher levels of osteocalcin when compared to cultured MSC. The rank order for osteocalcin synthesis was direct contact > indirect contract > MSC. The data indicate that in vitro direct contact between MSC and VEC induced osteogeneis.

### In vivo effect of VECs on MSC osteogenic potential

**Figure 3:**
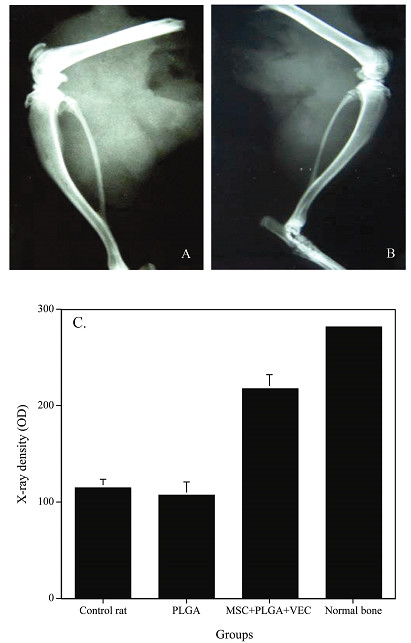
Soft X-ray images from right and left thigh and X-ray density analysis using NIH image software. A. Right thigh implanted with the PLGA seeded with the MSCs and VECs. B. Left thigh implanted with the PLGA seeded with the MSCs. C. X-ray density analysis. The data shown are the means ± SD from six experiments.

**Figure 4:**
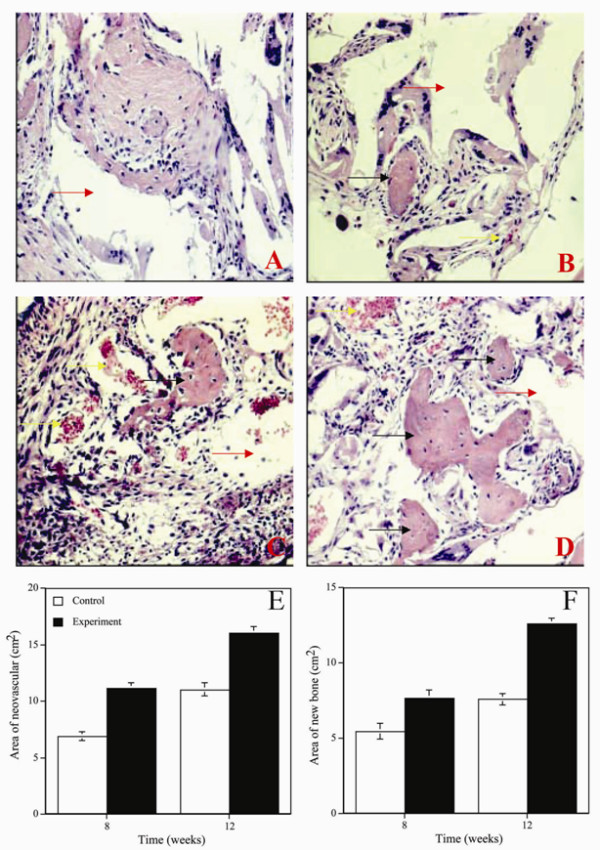
Hematoxylin and eosin staining of the implanted area from rat thigh and Quantitative measurements of new blood vessel and new bone from HE staining slides. A. Control group at 8 weeks post-implanted. B. Experiment group at 8 weeks post-implanted. C. Control group at 12 weeks post-implanted. D. Experiment group at 12 weeks post-implanted. Control is left thigh implanted with the PLGA seeded with the MSCs. Experiment is right thigh implanted with the PLGA seeded with the MSCs and VECs. Red arrow indicates PLGA material. Black arrow indicates new bone. Yellow arrow indicates blood vessel. E. New blood vessel. F. New bone. Control is left thigh implanted with the PLGA seeded with the MSCs. Experiment is right thigh implanted with the PLGA seeded with the MSCs and VECs. The data shown are the means ± SD from six experiments.

After 8 or 12 weeks following implantation specimens were collected and examined by soft X-rays. The corresponding radiographs are shown in figure 3. Eight weeks post-implantation, diffuse radio-opacity could be seen in the right thigh but not in the left (data not shown). This difference between the right thigh (with the MSC and VEC plated PLGA) and the left thigh (with the MSC plated PLGA) was even more pronounced at 12 weeks after the implantation (Fig. 3A and 3B). Densitometry of the various samples was performed using the NIH-image software and the results are displayed in figure 4. While the left thigh was no different from control, a 2-fold increase in density was seen in the right thigh (Fig. 3C). These results suggest that addition of VEC to cultured MSC induced osteogenesis in vivo.

Following soft X-ray analysis the samples were fixed in formalin and stained with hematoxylin & eosin. Typical histological pictures are presented in figure 4. Eight weeks after implantation, new bone tissue could be seen both in the left (Fig. 4A) and right (Fig. 4B) thighs. Four weeks later, new blood vessels were also found in both thighs. Histomorphometry of newly formed bone and blood vessels was also performed and the results are shown in figure 4E and 4F. Eight and 12 weeks after implantation, a significant (p < 0.05) increase both in neovasculature and bone formation could be seen in the right thigh (implanted with MSC and VEC-plated PLGA) when compared to the left thigh (implanted with MSC-plated VEC).

## Discussion

Angiogenesis, the development of vascular network, is essential for tissue growth and repair including bone [22,23]. Vascularization of implanted bone tissue can occur by in-growth of host blood vessels or to originate within the implanted bone tissue. In-growth of new blood vessels from the host progresses at a slow rate which is inadequate for large volumes of bone tissue. Regenerated bone containing potential blood vessel elements or blood vessel-like structures can facilitate the vascularization process [24,25]. These elements in tissue-engineered implants could be critical to their survival [22].

In the present study, we have demonstrated the use of direct contact or interaction between MSC and VEC for formation of vascularised bone tissue in vivo. Previously [26], we have shown that VEC could be successfully enriched under specific conditions. CD31- and von-Willebrand factor-positive VEC (data not shown) were cultured on PLGA discs. These VEC formed pre-vascular network-like structure on the surface of the PLGA (Fig. 1) and were also osteocalcin-negative and had very low ALP activity (Fig. 2). MSC are a good source for osteoprogenitors cells or osteostem cells which can differentiate to chrondogenic and osteogenic cells [27]. In vitro cultures of MSC-plated PLGA had low levels of ALP activity and of osteocalcin (Fig. 2). Addition of VEC either indirectly or directly, especially directly, to the MSC-plated PLGA increased the osteocalcin and ALP activity levels substantially (Fig. 2). In vitro direct contact of the MSC and VEC enhanced ~3.4 fold osteogenesis compared to MSC alone. These results indicated that the differentiation of MSC could be regulated not only by cytokines, but also by cell direct contact [12].

In vivo experiments demonstrated that MSC-plated PLGA implanted in the thigh muscle of the rat induced formation of new bone and blood vessels as seen in histological evaluation (Fig. 4). This newly formed bone could not be detected in soft X-ray analysis (Fig. 3). MSC and VEC-plated PLGA implanted in the rat's thigh led to substantially more vascularized bone tissue being formed (Fig. 4). In the case of MSC and VEC-plated PLGA newly formed bone could be seen in soft X-ray analysis (Fig. 3). The interaction of MSC and VEC is probably essential, complex and mutual since both new blood vessels and bone were generated. This effect of the MSC on the VEC is in accord with previous report which showed that hMSC could also secrete growth factors which stimulated endothelial cell proliferation [28-30].

In conclusion, our results demonstrate that VEC can enhance vascularization in engineered-bone tissue by the direct contact or interaction with MSC in the rat in vivo. This technique could be useful in repairing damaged bone tissue.
